# Single-Molecule-Level Quantification Based on Atomic Force Microscopy Data Reveals the Interaction between Melittin and Lipopolysaccharide in Gram-Negative Bacteria

**DOI:** 10.3390/ijms251910508

**Published:** 2024-09-29

**Authors:** Sheng Huang, Guoqi Su, Li Yang, Liangguang Yue, Li Chen, Jinxiu Huang, Feiyun Yang

**Affiliations:** 1 Animal Nutrition Institute, Chongqing Academy of Animal Science, Chongqing 402460, China; huangs@cqaa.cn (S.H.); sugq@cqaa.cn (G.S.); chenliyouxiang8@163.com (L.C.); 2 Institute of Nutrition and Feed, National Center of Technology Innovation for Pigs, Chongqing 402460, China; lekalter@163.com (L.Y.); 13021352791@163.com (L.Y.)

**Keywords:** melittin, lipopolysaccharide, atomic force microscopy, interaction force, kinetics characteristics, single-molecule force spectroscopy

## Abstract

The interaction forces and mechanical properties of the interaction between melittin (Mel) and lipopolysaccharide (LPS) are considered to be crucial driving forces for Mel when killing Gram-negative bacteria (GNB). However, how their interaction forces perform at the single-molecule level and the dissociation kinetic characteristics of the Mel/LPS complex remain poorly understood. In this study, the single-molecule-level interaction forces between Mel and LPSs from *E. coli* K-12, O55:B5, O111:B4, and O128:B12 were explored using atomic force microscopy (AFM)-based single-molecule force spectroscopy (SMFS). AFM-based dynamic force spectroscopy (DFS) and an advanced analytical model were employed to investigate the kinetic characteristics of the Mel/LPS complex dissociation. The results indicated that Mel could interact with both rough (R)-form LPS (*E. coli* K-12) and smooth (S)-form LPSs (*E. coli* O55:B5, O111:B4, and O128:B12). The S-form LPS showed a more robust interaction with Mel than the R-form LPS, and a slight difference existed in the interaction forces between Mel and the diverse S-form LPS. Mel interactions with the S-form LPSs showed greater specific and non-specific interaction forces than the R-form LPS (*p* < 0.05), as determined by AFM-based SMFS. However, there was no significant difference in the specific and non-specific interaction forces among the three samples of S-form LPSs (*p* > 0.05), indicating that the variability in the O-antigen did not affect the interaction between Mel and LPSs. The DFS result showed that the Mel/S-form LPS complexes had a lower dissociation rate constant, a shorter energy barrier width, a longer bond lifetime, and a higher energy barrier height, demonstrating that Mel interacted with S-form LPS to form more stable complexes. This research enhances the existing knowledge of the interaction micromechanics and kinetic characteristics of Mel and LPS at the single-molecule level. Our research may help with the design and evaluation of new anti-GNB drugs.

## 1. Introduction

Gram-negative bacteria (GNB), especially multidrug-resistant *E. coli* infections, are exponentially increasing and pose one of the most urgent global healthcare and economic threats [1]. A recent study showed that the total economic cost of antimicrobial resistance in pathogens such as *E. coli*, *Klebsiella pneumoniae*, *Acinetobacter baumannii*, and *Pseudomonas aeruginosa* has reached USD 2.8 billion per year in the United States [2]. Multidrug-resistant *E. coli* strains were the most common pathogen in the EU during 2015, resulting in over 30,600 infections and 9828 deaths [3]. According to the World Health Organization, Shiga-toxin-producing *E. coli* causes approximately 2.8 million cases of diarrhea and 3000 deaths every year worldwide [4]. These Gram-negative species have a structurally dynamic cell envelope, enabling them to resist multiple antibiotics [5]. However, new treatment methods have proven to be insufficient, and antimicrobial resistance has continued to escalate, acting like a global ticking time-bomb. There is an urgent need to develop new therapies to manage GNB infections and reduce the occurrence of bacterial resistance using new antibacterial drugs.

**Figure 1:**
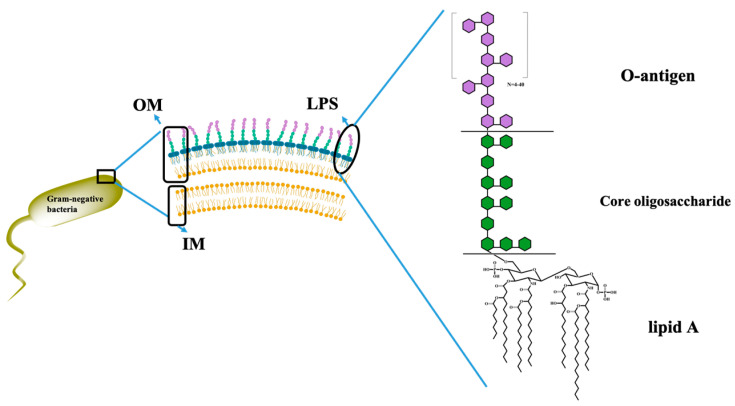
Schematic diagram of the cell membrane structure of GNB.

GNB are classified by their multilayered macromolecular structure on the cell envelope, which consists of three components. These are the outer membrane (OM), the peptidoglycan layer, and the inner membrane (IM) (Figure 1) [6]. The OM is a crucial component of GNB; it protects the bacteria from extreme external environments and acts as a barrier to arrest the entry of harmful compounds [7].

LPS is a critical component of the OM that maintains the structure and permeability of GNB [8]. LPS strongly interacts with each other through bridging with divalent cations that bind the negatively charged phosphate groups of the lipid A core, avoiding repulsion and maintaining OM integrity [9]. As LPS is the barrier that mediates the entry of antimicrobials, it has been increasingly recognized as a critical component in determining antimicrobial susceptibility and has become a target for antibacterial drug development. LPS typically consists of three structural regions: lipid A, a hydrophobic glycolipid which anchors LPS in the bacterial membrane; core oligosaccharide, a nonrepeating oligosaccharide which commonly contains sugars; and the O-antigen, a polysaccharide composed of multiple oligosaccharide repeating units (O units) each with two to seven residues from a broad range of common or rare sugars and their derivatives (Figure 1) [10]. The O-antigen structure offers the important property for the serotyping schemes of GNB and makes O serotyping one of the crucial roles in typing organisms for taxonomy [11]. The O-antigen is exposed on the cell surface and is usually highly immunogenic [12]. It is also a vital pathogenic factor and loss of O-antigen makes many pathogens serum-sensitive or causes other pathogenic impacts [13]. The O-antigen has also been shown to play a role in the resistance of bacteria to AMPs [14].

Antimicrobial peptides (AMPs) are promising antimicrobial reagents that could combat the ongoing global threat of multidrug-resistant bacteria [15]. It is generally agreed that AMPs kill bacteria via a membrane-lytic mechanism [16]. The membrane-targeted modes of action often lead to rapid killing. The majority of AMPs are amphiphilic and cationic peptides, whereas LPS has a net negative charge. AMPs are initially attracted to LPS by electrostatic interactions. With the increase in molecular AMPs, the electrostatic interaction and the penetration of AMP binding to the LPS are strengthened, and then AMPs diffuse and transfer into the IM of GNB [17]. AMPs have been inserted into the lysis cell membrane in transmembrane pore models and the nonmembrane pore models [18]. Hence, AMPs must first interact with and penetrate the LPS before reaching the cytoplasm of the GNB [19]. Therefore, LPS is the foremost site of interaction with the AMPs.

Melittin (Mel) is a significant AMP. It is found in bee venom, and multiple in vitro studies have observed that Mel demonstrates substantial antimicrobial activity against GNB [20,21]. Our previous research revealed that Mel showed a rapid bactericidal effect by interacting with LPS and penetrating the bacterial OM and IM of *E. coli* ATCC 25922 [22]. Brand et al. showed that Mel interacted with an OM model of GNB, increasing the membrane permeability and decreasing OM stability when bound to Mel [23]. Structural and thermodynamic research has indicated that the binding of Mel to LPS is an endothermic process, and Mel’s helical conformation and C-terminus could be significant factors when recognizing the LPS [24]. However, the above-mentioned studies only used conventional bulk scales and did not clarify the single-pair biomechanical interaction between Mel and LPS at a micro- or nanoscale. A more profound, single-molecule-level quantification of the interactions between Mel and LPS could help to elaborate Mel’s mechanism of antimicrobial activity, leading to the development of new anti-GNB drugs.

Atomic force microscopy (AFM) is regarded as an effective method to quantify biomolecular interactions at the nanoscale level [25]. Sun et al. used AFM to reveal the interaction force between a pro-apoptotic protein (BAX) and B-cell lymphoma 2 (Bcl-2). The results showed complex non-covalent interactions at the BAX/Bcl-2 site when using AFM-based single-molecule force spectroscopy (SMFS) [26]. Another study used AFM to evaluate the interaction forces between LPS and monoclonal antibodies (mAbs). The results indicated that a single bond between LPS and mAbs included a non-specific component as well as an immunochemically specific component [27]. Previously, we probed the change in the calcium ion (Ca^2+^)-induced interaction forces between Mel and calmodulin using AFM [28]. The results indicated that the unbinding force between Mel and calmodulin increased in the presence of Ca^2+^ in a concentration-dependent manner.

This study used AFM to quantitatively describe the interactions between Mel and LPSs at the single-molecule level. The appropriate analysis model determined the contribution of non-covalent bonds to the Mel/LPS interaction. We also portrayed the dissociation kinetic characteristics of the Mel/LPSs complex through AFM-based DFS. The results provide new insight into biomechanical information about the LPS/Mel interaction and present a novel approach to exploring the bactericidal mechanism of Mel.

## 2. Results and Discussion

### 2.1. Morphological Characterization of LPS Using Different E. coli Strains

**Figure 2:**
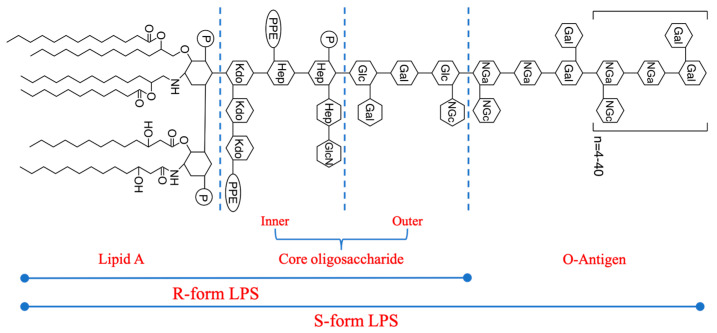
Chemical structure of LPS. Kdo: 3-deoxy-α-Dmanno-oct-2-ulopyranosonic acid; PPE: phosphatidyl ethanolamine; Hep: heptose; Glc: glucose; Gal: galactose; NGc: glucosamine; NGa: galactosamine; P: phosphate; R: rough; S: smooth.

LPS is a significant glycolipid substance composed of three structural domains. These are lipid A, the core oligosaccharide (Core OS), and the O-antigen (Figure 2) [29,30]. The overall structure of LPS is conserved, and the lipid A structure is conserved at the species level. The Core OS varies among species and even between certain strains of one species. The most diverse component of LPS is the O-antigen. Not only do the structure and composition differ within a species at the strain level (leading to a high number of serotypes of *E. coli* [31]), but also certain Gram-negative bacteria do not synthesize the O-antigen. Molecules composed of only lipid A and the Core OS are typically referred to as a “rough (R) form” LPS as opposed to a “smooth (S) form” LPS, which includes the O-antigen [32]. The LPSs from *E. coli* O55:B5, O111:B4, and O128:B12 used in this study were typical S-form LPSs and the LPS from *E. coli* K-12 was an R-form LPS.

**Figure 3:**
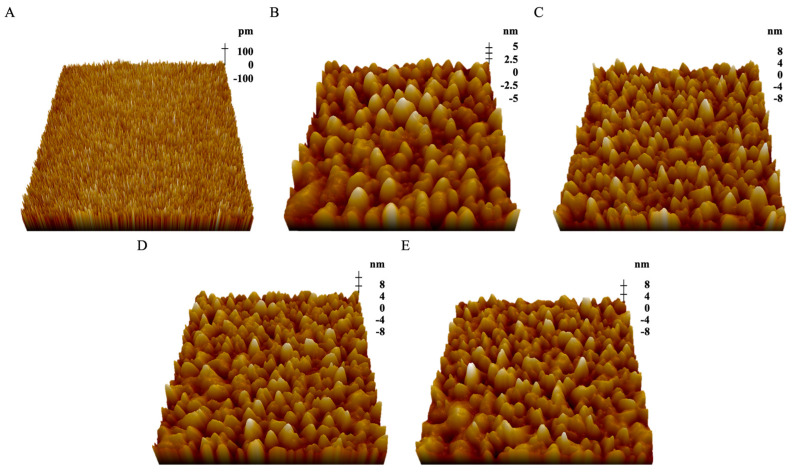
AFM images of the 3D topography surface of mica and LPSs from different *E. coli strains*. (**A**) Mica; (**B**) LPS from *E. coli* K-12; (**C**) LPS from *E. coli* O55:B5; (**D**) LPS from *E. coli* O111:B4; (**E**) LPS from *E. coli* O128:B12. The samples were scanned using a line frequency of 1 Hz with a resolution of 512 × 512 pixels and a scanning size of 1 × 1 μm.

In this work, we immobilized the LPS samples onto the mica surface using a COOH-NH_2_ linking method. The non-contact mode of AFM was used to reveal the nanoscale surface morphology of the mica and LPSs. The surface of the fresh mica was smooth and burr-free (Figure 3A). The LPS molecules were evenly distributed, and most of the imaged LPS particles were nanosized aggregates (Figure 3B–E).

The surface morphology of the mica and LPSs was analyzed using XEI software to quantitatively characterize the surface morphology characteristics. The average roughness (Ra) and average height (μ) are used to describe the surface properties of the mica and LPSs (Table 1). The results revealed that the average roughness of the fresh mica was 0.057 ± 0.003 nm^2^, with an average height of 0.231 ± 0.004 nm. This suggested that the mica had a very smooth surface. After covalently linking the LPS from *E. coli* K-12 onto the mica surface, the average surface roughness significantly increased to 0.216 ± 0.004 nm^2^, with an average height of 13.245 ± 0.071 nm. This indicated that the distribution of the LPS was single-layered and prone to assembly on the surface in bundles.

When the LPSs from *E. coli* O55:B5, O111:B4, and O128:B12 were anchored onto the mica, the average roughness and average height of the three LPSs did not show significant differences. The average roughness and average height of the three LPSs significantly improved compared with the LPS from *E. coli* K-12. This result was consistent with a previous description [27]. The O-antigen portion of the LPSs from *E. coli* O55:B5, O111:B4, and O128:B12 had a long hydrophilic chain. There was less water in the hydrophilic chain because the AFM imaging was performed in air. This caused the hydrophilic chain to be closer. This type of hydration caused the surface of the LPSs from *E. coli* O55:B5, O111:B4, and O128:B12 to show a higher average roughness and average height than the LPS from *E. coli* K-12. Hydration impacted the LPS from *E. coli* K-12 less because it lacked the O-antigen portion [33]. The experimental results indicated that LPS could successfully be anchored onto the surface of mica using COOH–NH_2_ linking. This laid the foundation for the subsequent mechanical detection.

### 2.2. Probing the Interaction Forces between Mel and LPS

In this study, we focused on investigating the single-molecule interaction force between Mel and LPS. It was difficult to dynamically study the interaction biomechanics between Mel and LPS in a homogenous solution unless they were fixed to a matrix surface. We used a self-assembled monolayer (SAM) method to adhere Mel onto the gold-modified AFM probe surface, as previously described [28]. The LPS samples were anchored onto the mica surface using the process described by Ananchenko et al. [27]. The detection of the interaction forces between Mel and LPS was enhanced using AFM-based SMFS.

**Figure 4:**
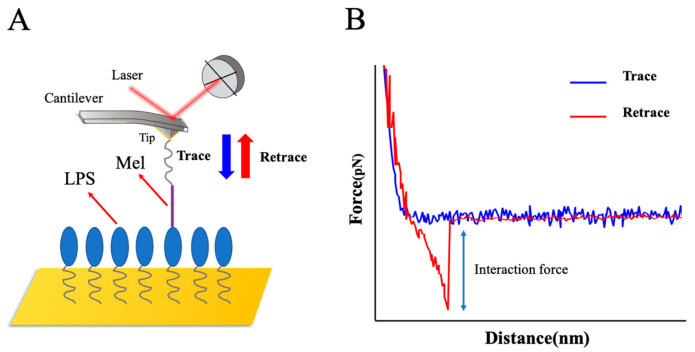
Detection of adhesion forces between Mel and LPS using AFM-based SMFS. (**A**) The principle of the experimental process. An AFM probe functionalized with Mel was moved toward and retracted from a mica surface functionalized with LPS. (**B**) Representative force-distance curve showing the force data obtained using the AFM.

The force–distance curves were recorded after the Mel-modified probes were moved to a site on the surface of the LPS and then retracted to an initial set point. When the probes approached the set point on the surface of the LPS and were retracted from the binding point, the probes were deflected because of the interaction forces between Mel and LPS and the instrument produced a “force–displacement” curve. A typical operation process and a Mel/LPS force–distance curve are shown in Figure 4.

The AFM probes could be regarded as an elastic material. The deflection of the probes was converted to the force (*F*) according to Hooke’s law, as follows:

(1) F=k×∆z

where ∆z is the deflection of the probes, and *k* is the spring constant of the probes.

**Figure 5:**
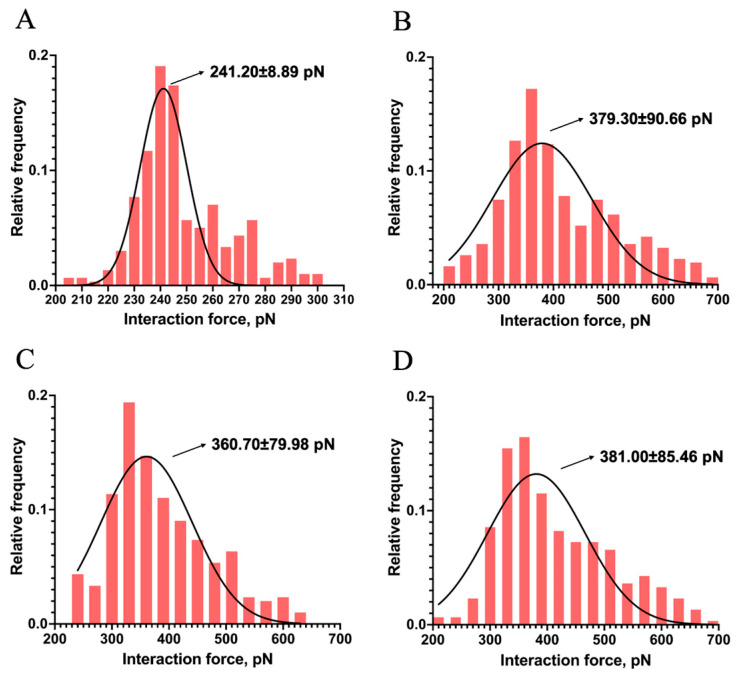
Interaction force–frequency distribution histograms and Gaussian fitting curves for Mel and LPS samples from *E. coli* K-12 (**A**), *E. coli* O55:B5 (**B**), *E. coli* O111:B4 (**C**), and *E. coli* O128:B12 (**D**).

The binding of Mel and LPS was random during the interaction force measurement procedure. Interaction forces did not occur in each measurement because of the electrostatic interaction between the probes and mica [34]. The interaction forces between Mel and LPS were measured several hundred times to overcome this limitation. Force histograms were drawn, with approximately 300 force measurements for each Mel and LPS group. The possible interaction forces were fitted using a Gaussian distribution, as shown in Figure 5.

The maximum interaction force between Mel and LPS from *E. coli* K-12 appeared at 241.20 ± 8.89 pN (Figure 5A). The maximum interaction forces between Mel and LPS from *E. coli* O55:B5, O111:B4, and O128:B12 occurred at 379.30 ± 90.66, 360.70 ± 79.98, and 381.00 ± 85.46 pN, respectively (Figure 5B−D). The results indicated that Mel could interact with both the R-form LPS (*E. coli* K-12) and the S-form LPSs (*E. coli* O55:B5, O111:B4, and O128:B12). The S-form LPS showed a greater interaction with Mel than the R-form LPS. Slight differences existed in the interaction forces between Mel and the diverse S-form LPSs. The results suggested that both the polysaccharide and lipid A structures of LPS contributed to the interaction between Mel and LPS.

### 2.3. Investigating the Single-Molecule Interaction between Mel and LPS

The results of the interaction forces obtained during the AFM force detection reflected the interaction between multiple pairs of Mel and LPS because the AFM probe tip had a certain radius of curvature. This observation was in agreement with a previous study [28]. The Poisson statistical analysis method was used to explore the interaction between a single Mel and LPS pair and to analyze the measured interaction forces [35,36]. Before proceeding with the analysis, we assumed that the total interaction forces in an assigned area measured by AFM consisted of a finite distribution of the number of interacting bonds, and were subject to a Poisson distribution. The specific interaction force (*F*) could be used to derive the specific interaction force (*F_i_*) and non-specific interaction force (*F*_0_) of a single Mel/LPS pair, using the method previously published [27].

As described by the Poisson distribution, the mean value of and variance in the bond-break number (*n*) of Mel/LPS are equal:

(2) $$\mu_{n}=\delta_{n}^{2}$$

The *F* measured from an actual AFM measurement was related to the bond-break number (*n*) in a single trace and retrace event, as follows:

(3) $$F=nF_{i}$$

In the equation, *F* is the mean value of a single bond-breaking force and is expected to be a constant value.

The variance ($\sigma_{m}^{2}$) and mean (*μ_m_*) values of the interaction forces were acquired by detecting multiple trace and retrace events. The following formula were calculated because of the relationship between the measured interaction forces and the bond-break number (*n*):

(4) $$\mu_{m}=\mu_{n}F_{i}$$

(5) $$\sigma_{m}^{2}=\sigma_{n}^{2}F_{i}^{2}$$

According to Formulas (4) and (5), the single-pair interaction force (*F_i_*) could then be calculated using the following formula:

(6) $$F_{i}=\frac{\sigma_{m}^{2}}{\mu_{m}}$$

When considering the possible non-specific interaction force (*F*_0_), Formulas (4) and (5) were changed to

(7) $$\mu_{m}=\mu_{n}F_{i}+F_{0}$$

(8) $$\sigma_{m}^{2}=\sigma_{n}^{2}F_{I}^{2}=\mu_{m}F_{i}\;-F_{i}F_{0}$$

According to the Poisson distribution model, the specific interaction force (*F_i_*) and the non-specific interaction force (*F*_0_) between a single Mel/LPS pair could then be calculated using the following equation:

(9) $$\sigma_{m}^{2}=\mu_{m}F_{i}-F_{i}F_{0}$$

where $\sigma_{m}^{2}$ and *μ_m_* are the variance and mean values of the detected interaction forces, respectively.

**Figure 6:**
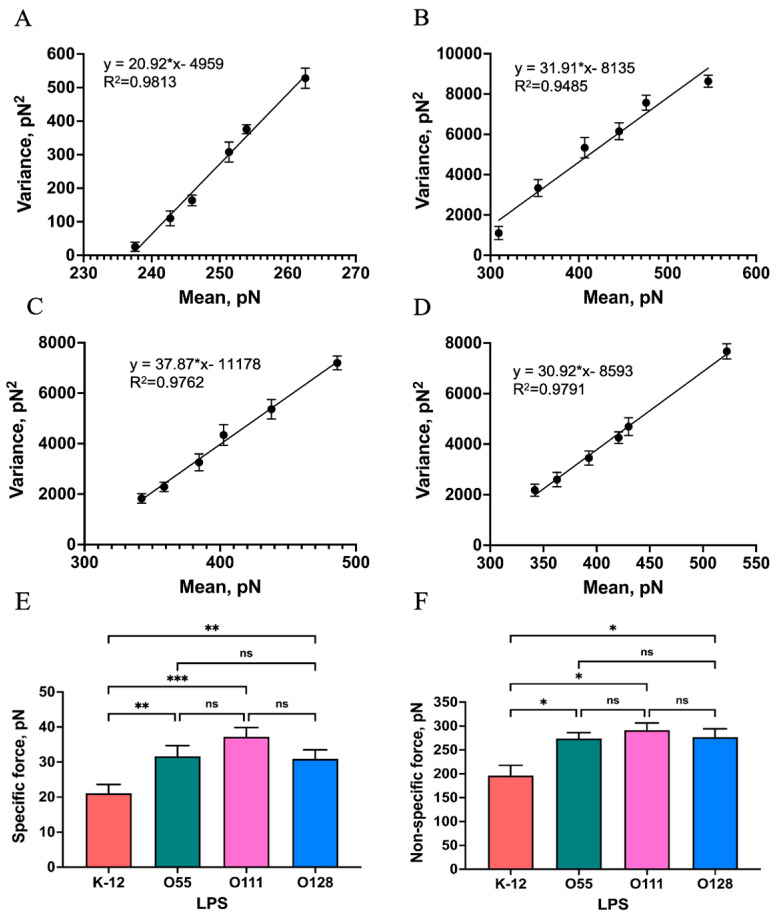
Measurements of single-molecule specific and non-specific interactions between Mel and LPS. (**A**–**D**) Linear relationships of the mean versus the variance in the interaction forces between Mel and LPS from *E. coli* K-12, O55:B5, O111:B4, and O128:B12, respectively. Specific (**E**) and non-specific (**F**) interaction forces between a single Mel and LPS pair (ns: no significance. * *p*-values < 0.05, ** *p*-values < 0.01, and *** *p*-values < 0.001 from an ordinary one-way ANOVA).

The total interaction forces between Mel and LPS were measured using 50~60 repeats at six randomly chosen points on the LPS surface. The variance ($\sigma_{m}^{2}$) and mean (*μ_m_*) values of the force measurements were calculated approximately 300 times. The linear fitting of the variance versus the mean is shown in Figure 6. The slope and the intercept values of the obtained regression curves were the *F_i_* and the *F_i_F*_0_ of the single-molecule pair of Mel and LPS, respectively.

An earlier study addressed the role of LPS in regulating the interaction with AMPs. The O-antigen of LPS could bind to organic molecules by electrostatic and hydrogen bonding [37]. Wen et al. indicated that the electrostatic interaction could regulate the AMP–LPS vesicle interaction [38]. Joshua et al. suggested that the interaction between immobilized AMPs and deliquescent LPS molecules occurs in the lipid A portion because of electrostatic attraction and hydrophobic interactions [39]. A simulation study of molecular dynamics revealed that the Gly1 residue in the N-terminus of Mel interacts with the phosphate group in lipid A by formatting the hydrogen bonds [19]. The above studies indicated that hydrogen bonding as well as hydrophobic and electrostatic interactions play crucial roles in the interaction between AMPs and LPS. In a Poisson distribution analysis, the chemical and hydrogen bonds contribute to the specific interaction, whereas the electrostatic and hydrophobic interactions are included in the non-specific interaction. In this study, we resolved the specific and non-specific forces between a single Mel and LPS pair using the Poisson distribution. As shown in Figure 6E,F, the specific interaction forces between Mel and the LPSs from *E. coli* K-12, O55:B5, O111:B4, and O128:B12 were 21.10 ± 2.08, 31.70 ± 2.51 37.20 ± 2.14, and 30.90 ± 2.07 pN, respectively. The non-specific interaction forces were 196.30 ± 30.59, 273.90 ± 17.6, 291.40 ± 21.29, and 276.80 ± 24.62 pN, respectively. These results indicated that Mel could bind to the LPS from *E. coli* K-12 by hydrogen bonds, electrostatic attraction and hydrophobic interactions, simultaneously showing the specific and non-specific interaction forces. Mel interacting with the S-form LPS samples showed greater specific and non-specific interaction forces than the R-form LPS (*p* < 0.05), because of the existence of the O-antigen (Figure 6E,F). There was no significant difference in the specific and non-specific interaction forces among the three S-form LPSs (*p* > 0.05). This proved that the presence of the O-antigen could promote the interaction between Mel and LPS. The O-antigen variability might not affect the interaction between Mel and LPS. The research results of connor et al. showed that the insensitivity of uropathogenic *E. coli* sequence type 131 (ST131) to colicin was dependent on the presence of O-antigen. Re-introduction of O-antigen into *E. coli* K-12 made it again insensitive to colicin, demonstrating that O-antigen in LPS plays a critical role in the sensitization and interaction of *E. coli* toward colicin [40]. The apparent differences in the interaction forces between melittin and S-LPS and R-LPS in this study also indicated the importance of O-antigen in Mel and LPS interactions. We preliminarily speculated that Mel first generated a hydrogen bond and an electrostatic interaction with the O-antigen in the process of LPS binding, and then the amino acids of Mel bound with the lipid A and core polysaccharide modules via hydrogen bonds, an electrostatic interaction, and a hydrophobic interaction. In addition, the contribution of lipid A and core polysaccharide modules in the interaction between LPS and Mel deserves further investigation.

Due to LPS being the initial contact site for AMPs, its structure may be crucial for the sensitivity of GNB to AMPs [41]. However, the mode of action of AMPs will determine whether a stronger affinity for LPS contributes to or impedes membrane disruption. Although the initial interaction between AMPs and LPS is believed to be beneficial for AMP function, it also indicated that LPS can actually inhibit the antibacterial function of AMPs. It can achieve this by strongly binding to AMPs, thereby preventing entry into the bacterial inner membrane. It was shown for *E. coli* that the addition of isolated LPS protects the bacteria from killing by CATH-2, PMAP-36, and LL-37 [42]. In this study, there was a significant difference in the interaction between R-LPS and S-LPS, while there was no significant difference among the three selected S-LPSs. This requires expanding the scope of LPS in subsequent studies and further validating the results of this study in situ on *E. coli*. The effects of LPS on the antibacterial activity of Mel could be elucidated by combining interaction mechanics and antibacterial activity research.

### 2.4. Quantitatively Probing the Kinetic Characteristics of the Mel/LPS Dissociation

Our study showed that Mel and the LPSs from different *E. coli* samples presented a strong interaction, but there were a few differences. We explored whether these differences stemmed from the dissociation process of the Mel/LPS complex using DFS-based AFM. The dissociation kinetic characteristics between Mel and LPS were obtained and extracted by varying the loading rate (LR) and recording the interaction forces during the DFS experiments [43].

**Figure 7:**
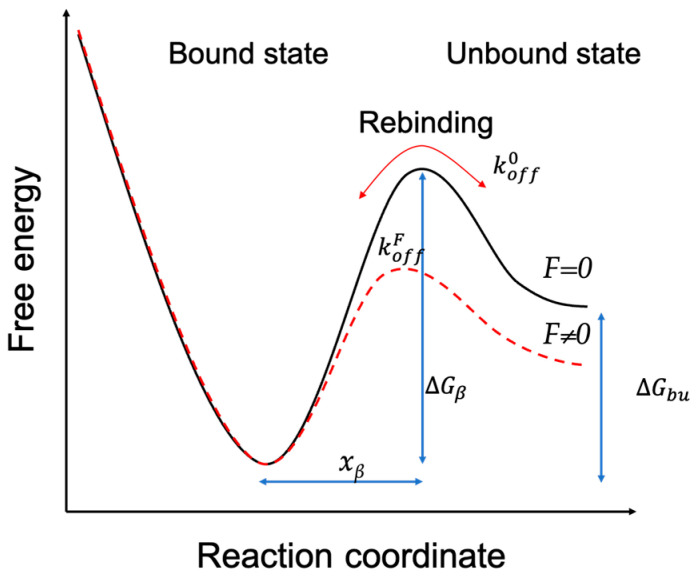
Schematic diagram of the energy landscape, depending on external forces. The Bell–Evans model describes the Mel/LPS bond as a two-state model. The bound state is separated from the unbound state by a single energy barrier located at a distance (*x_β_*).

The Bell–Evans model (Figure 7) was used to express the relationship between the interaction force (*F*) and the logarithm of the LR (*r*), as follows:

(10) $$F=\frac{k_{B}T}{x_{\beta }}In\left(\frac{{rx}_{\beta }}{k_{off}k_{B}T}\right)$$

where *F* is the most probable interaction force, *k_B_* is Boltzmann’s constant, *T* is the absolute temperature, *x_β_* is the energy barrier width, *r* is the LR, and *k_off_* is the dissociation rate constant.

The bond lifetime (*τ_off_*) of the single Mel and LPS pair binding was the inverse form of the dissociation rate constant (*k_off_*), as follows:

(11) $$\tau_{off}=\frac{1}{k_{off}}$$

**Figure 8:**
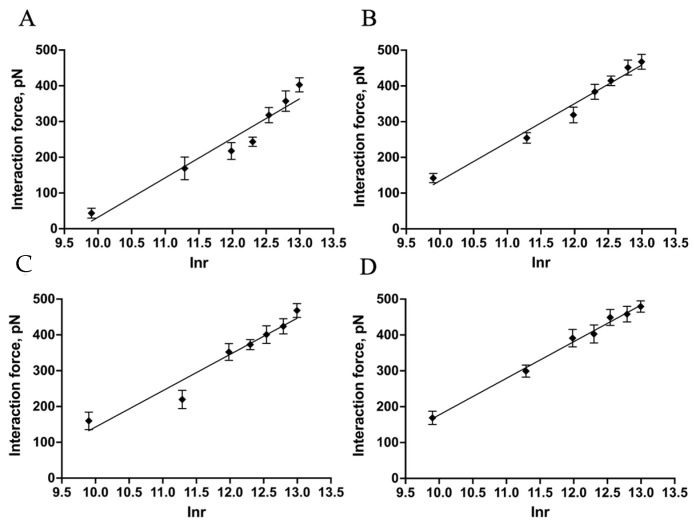
Probing the kinetic characteristics of the Mel/LPS dissociation. DFS plot showing the distribution of the interaction forces as a function of their LR measured between Mel and the LPSs from *E. coli* K-12 (**A**), O55:B5 (**B**), O111:B4 (**C**), and O128:B12 (**D**).

In the experiment, the force-distance curves were gathered under a gradient increasing LR, and the most probable interaction forces confirmed by Gaussian distribution were plotted against the LR on a log scale (Figure 8).

Several AFM-based DFS studies have shown that the interaction between biological macromolecules is related to an intrinsic interaction and the LRs. Using DFS, it is possible to transform the mechanics information into thermodynamic and kinetic parameters [44,45,46]. Detecting the most probable interaction forces at various LRs revealed the dissociation dynamics of the Mel/LPS interaction. The results showed that the most probable interaction forces (as a function of the LR) exhibited only one regression curve (Figure 8), which was the same in earlier studies [47,48]. This indicated that the binding complex of Mel/LPS followed a simple two-state model in which the bound state was separated from the unbound state by a single energy barrier [49]. Once the *k_off_* had been obtained, the height of the energy barrier, ∆G, could be deduced using the following equation:

(12) $$∆G=-k_{B}TIn\frac{k_{off}h}{k_{B}T}$$

where *h* is Planck’s constant and *k_B_T* is the thermal energy.

An early study of the Cytochrome C 551 and Azurin interaction using AFM-based SMFS showed that the dissociation rate constant (*k_off_*) of the Cytochrome C 551–Azurin complex was 14 S^−1^ [50]. Another study investigated the mechanistic stability of Anabaena ferredoxin-NADP + Reductase (FNR) with its redox protein partners ferredoxin (Fd) and flavodoxin (Fld) using DFS of AFM. The results showed that the *k_off_* values of FNR/Fd and FNR/Fld are 21.2 and 55.7 S^−1^, respectively [51]. The values of the dissociation rate constant (*k_off_*) were 110.91 ± 5.51, 108.04 ± 4.27, 99.82 ± 5.57, and 101.44 ± 3.54 S^−1^ for Mel and the LPSs from *E. coli* K-12, O55:B5, O111:B4, and O128:B12, respectively (Table 2). Compared with the *k_off_* value of Mel/R-LPS, the *k_off_* values of the three Mel/S-LPS samples were smaller. This demonstrated that the Mel and S-LPS interactions were stronger than Mel and R-LPS, probably owing to the lower stability of the Mel/R-LPS complex. Our results preliminarily indicated that the stability of Mel/LPS complexes was lower than that of complexes formed by protein–protein interaction, but higher than the electron transfer protein, with typical *k_off_* values up to 10^3^ S^−1^ [52]. The values of the energy barrier width (*x_β_*) for Mel and the LPSs from *E. coli* K-12, O55:B5, O111:B4, and O128:B12 were 0.1052 ± 0.0018, 0.0946 ± 0.0003, 0.0872 ± 0.0003, and 0.0842 ± 0.0003 nm, respectively. The energy barrier widths (***x_β_***) were shorter for three Mel/S-LPS samples compared with Mel/R-LPS. This indicated that the energy landscape could be described as a narrower energy valley [53], with less conformational variability [54]. The bond lifetime (*τ_off_*) values of the binding of the single Mel/LPS pair were 9.038 ± 0.448, 9.271 ± 0.371, 10.005 ± 0.574, and 9.870 ± 0.349 ms, respectively. The single-pair Mel/R-LPS bond lifetime (*τ_off_*) was shorter than three Mel/S-LPS samples. This indicated the high affinity and stability of the Mel/S-LPS complex, with a longer lifetime for high-affinity interactions. The heights of the energy barrier (∆*G*) values for Mel and the LPSs from *E. coli* K-12, O55:B5, O111:B4, and O128:B12 were 24.748 ± 0.049, 24.774 ± 0.040, 24.854 ± 0.056, and 24.837 ± 0.035 *k_B_T*, respectively. The calculation of the energy barrier height revealed that the ∆*G* values of Mel and S-LPS were higher than Mel and R-LPS, suggesting that the Mel/S-LPS complexes were more challenging to dissociate than Mel and R-LPS [53].

## 3. Materials and Methods

### 3.1. Materials and Reagents

LPSs from *E. coli* O55:B5, *E. coli* O111:B4, *E. coli* O128:B12, and *E. coli* K-12 were purchased from Sigma-Aldrich (Shanghai, China). Melittin was purchased from Yuanye (Shanghai, China). Phosphate-buffered saline (PBS) (1×; pH 7.2–7.4), N-hydroxysuccinimide (NHS), N-(3-dimethylaminopropyl)-N-ethylcarbodiimide hydrochloride (EDC), and 16-mercaptohexadecanoic acid (MHA) were obtained from Sigma-Aldrich (Shanghai, China). (3-aminopropyl)triethoxysilane (APTES), triethylamine, and ethanolamine were purchased from Macklin (Shanghai, China). AFM probes (OMCL-AC240TS and PPP-CONTCSAu) were purchased from Olympus (Tokyo, Japan) and Nanosensors (Neuchatel, Switzerland). Gold-coated silicon substrates and mica were purchased from Ted Pella (Redding, CA, USA). Deionized water (18.25 MΩ·cm) was obtained using a Direct-Q 3 Millipore Ultrapure water purification system (Burlington, MA, USA).

### 3.2. Immobilization of Mel on Gold-Coated AFM Probes

The immobilization of Mel on gold-coated AFM probes was implemented as previously described, but with a simple modification [28]. Briefly, gold-coated AFM probes (PPP-CONTCSAu, with a tip radius < 50 nm, a resonance frequency of 25 kHz, and a standard spring constant of 0.2 N/m) were UV-radiated for 15 min to remove contamination from the surface. After cleaning, the probes were immersed in 1 mM of an MHA ethanolic solution for 24 h at room temperature. This was followed by washing with ethanol and drying with nitrogen. Subsequently, the MHA-modified probes were immersed in a mixture solution of NHS (10 mg/mL) and EDC (25 mg/mL) and incubated for 30 min at room temperature to activate the terminal carboxyl groups. Finally, the treated probes were inserted into the Mel solution (10 µg/mL in PBS) and incubated overnight at 4 °C. The remaining activated carboxyl groups were deactivated using 1 M ethanolamine. After washing with PBS, the Mel-functionalized probes were used for the force spectroscopy measurements.

### 3.3. Immobilization of LPSs on Mica

A previously described method with a modification was used to functionalize the LPS samples onto mica [27]. First, a vacuum desiccator was filled with argon, and freshly cleaved mica was placed inside. Next, 30 μL APTES and 10 μL triethylamine were placed into the vacuum desiccator for mica-amination within 1.5 h. The aminopropyl-modified mica samples were used for the further immobilization of the LPS. A mixture of the PBS solution of NHS (final concentration of 10 mg/mL) and EDC (final concentration of 25 mg/mL) was sequentially added to the LPS samples (final concentration of 10 μg/mL) and PBS solution. The mixture was stored for 1 min, and then the aminopropyl-modified mica samples were immersed in this solution. After 2 h incubation, the mica samples were thoroughly washed using PBS for the subsequent morphological imaging and force spectroscopy measurements.

### 3.4. Interaction force measurements for Mel and LPS

The adhesion forces between Mel and LPS were measured using Park System NX-10 atomic force microscopy (Suwon, Korea), and an earlier research method with modifications [55]. The Mel-functionalized AFM probes were first scanned across the LPS monolayer and then the bare mica (as a blank control) at a randomly selected location. Force measurements were then obtained. The Mel-functionalized probes were positioned toward the desired location on the surface of the LPS and then retracted back to the initial point. The probes were deflected after they approached the monolayer surface and then retracted to the binding point because of the Mel/LPS interaction force. This was detected as a “voltage-displacement” signal on the instrument; it was then transformed into a “force–displacement” curve. Mel-modified probes were used for all force experiments in this study. The spring constant was calibrated using the thermal fluctuation method.

All interaction force measurements for Mel and LPS were performed using a contact mode at 25 °C. Several hundred force curves were collected from three experiments (~300 force curves per set) using three different samples and three probes. The velocity of the probe during retracement was set to 1000 nm/s. Interaction forces were calculated and analyzed from the collected “force–displacement” curves using the XEI processing software program (Suwon, Korea). Six locations in the LPS monolayer were randomly selected. Measurements were obtained approximately 50 to 60 times from each point to obtain a more accurate statistical analysis. LPS-free mica was used as the control group. A block study was conducted by measuring the interaction forces in the presence of free Mel in a PBS solution.

### 3.5. Dynamic Force Spectrum Measurements for Mel and LPSs

AFM-based DFS was used to measure the interaction forces to estimate the dissociation kinetic parameters between Mel and LPSs. The basic operation referenced an earlier study [49] with a modification. Briefly, the interaction forces between Mel and LPSs were measured using the same approaching velocity of 1000 nm/s and a variable retraction velocity range from 100 to 2200 nm/s. The threshold force was 500 pN and the probe contact time remained at 0.6 s. All sample experiments were performed at least three times. All force curves were analyzed using the XEI processing software program (Suwon, Korea).

### 3.6. Morphological Characterization of LPS

All morphological images of LPS were obtained using Park System NX-10 atomic force microscopy (Suwon, Korea) and an approach, as described earlier, with a minor modification [56]. The AFM imaging study was performed at a scan rate of 0.5~1 Hz, with the image resolution set to 512 × 512. Briefly, AFM was performed in a non-contact mode in air with a scanning size of 1 × 1 μm. Images of the LPS samples and the roughness of the surfaces were collected and analyzed using the XEI processing software program (provided by the manufacturer).

### 3.7. Data Analysis

The statistical analysis was performed using GraphPad Prism 10 software. The force data were shown as the mean with a standard deviation (SD) from at least three independent experiments. Statistical significance was analyzed using a one-way ANOVA. Statistical differences were expressed as *** *p* < 0.0001, ** *p* < 0.001, and * *p* < 0.005.

## 4. Conclusions

In this study, we used AFM-based SMFS and DFS methods to quantify and reveal the interaction between Mel and LPS at the single-molecule level. The interaction forces between Mel and LPS were detected by approximating Mel-modified probes to an LPS monolayer and then retreating to the set original position. We observed that Mel could interact with both R-form LPS and S-form LPS. The S-form LPS showed a stronger interaction with Mel than the R-form LPS. A Poisson statistical analysis method was used to analyze the specific and non-specific interaction forces between Mel and LPS at the single-molecule level. The results revealed that Mel interacted with S-form LPS with greater specific and non-specific interaction forces than the R-form LPS because of the existence of the O-antigen. This demonstrated that Mel could interact with the O-antigen in the process of LPS binding by generating a hydrogen bond and electrostatic attraction. The amino acids of Mel bound with lipid A using hydrogen bonds, electrostatic attraction, and a hydrophobic interaction. We also explored the dissociation process of the Mel/LPS complex using DFS-based AFM to reveal the reason for the variations in the interaction forces between Mel and different LPSs. An analysis of the Bell–Evans model revealed that the complex formed by Mel and the S-form LPS had a shorter energy barrier width and a smaller dissociation rate constant during the dissociation process. This indicated that Mel could form a more stable complex after interacting with the S-form LPS. Future studies should quantitatively assess the interaction between Mel and LPS using *E. coli* in situ to verify the results of this study and further clarify the bactericidal and bacteriostatic activity mechanisms of Mel. In summary, this research is a complement to the traditional screening of AMPs based on antibacterial biological activity, elucidating the antibacterial mechanism from a biomechanical perspective and providing an exciting insight into the use of AFM.

## Figures and Tables

**Table 1:** Average roughness (Ra) and average height (μ) of the mica and LPSs.

No.	Sample	Parameter	
		Ra, nm^2^	μ, nm
1	Mica	0.057 ± 0.003	0.231 ± 0.004
2	LPS from *E. coli* K-12	0.216 ± 0.004	13.245 ± 0.071
3	LPS from *E. coli* O55:B5	0.304 ± 0.002	17.352 ± 0.045
4	LPS from *E. coli* O111:B4	0.335 ± 0.007	18.106 ± 0.062
5	LPS from *E. coli* O128:B12	0.315 ± 0.011	17.566 ± 0.051

**Table 2:** Kinetic and energy landscape parameters of the Mel/LPS dissociation.

Sample	*K_off_*; S^−1^	*x_β_*; nm	τ*_off_*; ms	∆*G*; *k_B_T*
LPS from *E. coli* K-12	110.91 ± 5.51	0.1052 ± 0.0018	9.038 ± 0.448	24.748 ± 0.049
LPS from *E. coli* O55:B5	108.04 ± 4.27	0.0946 ± 0.0003	9.271 ± 0.371	24.774 ± 0.040
LPS from *E. coli* O111:B4	99.82 ± 5.57	0.0872 ± 0.0003	10.005 ± 0.574	24.854 ± 0.056
LPS from *E. coli* O128:B12	101.44 ± 3.54	0.0842 ± 0.0003	9.870 ± 0.349	24.837 ± 0.035

## Data Availability

Data are contained within the article.
